# Dissipation of reactive inhibition is sufficient to explain post-rest improvements in motor sequence learning

**DOI:** 10.1038/s41539-022-00140-z

**Published:** 2022-10-06

**Authors:** Mohan W. Gupta, Timothy C. Rickard

**Affiliations:** Department of Psychology, University of California, San Diego, CA USA

**Keywords:** Human behaviour, Consolidation

## Abstract

The prevailing hypothesis for observed post-rest motor reaction time improvements is offline consolidation. In the present study, we present evidence for an alternate account involving the accrual and dissipation of reactive inhibition. Four groups of participants (*N* = 159) performed a finger-tapping task involving either massed (30 s per trial) or spaced (10 s per trial) training, and with one of two break intervals between each trial: 10 s or 30 s. After 360 s of training in each group, there was a 300 s rest period followed by a final test on the same task. The results show that the smaller the ratio of break time to on-task trial time during training, the larger the improvement in reaction time after the rest period. Those results are fully consistent with a model that assumes no facilitating offline consolidation, but rather learning that is concurrent with performance and reactive inhibition that builds during performance and dissipates during breaks.

## Introduction

A fundamental question about motor learning is whether it occurs online (concurrently with performance) or offline (during break periods). Pertinent to that question is the repeated observation that after a rest period – whether involving sleep, five minutes, or even 10 s – there are reaction time (RT) improvements in motor sequence tasks^1–8^. Numerous authors have concluded that those improvements are due to a form of offline memory consolidation that enhances learning and results in superior behavioral performance, rather than merely stabilizing memory as in the case of declarative learning^1–3,5,7,8^. However, the consolidation account is unable to explain several phenomena across different rest time scales. First, in sleep studies that adequately controlled for various factors like circadian rhythms and reactive inhibition (RI; the slowing of RT as one continuously performs a motor task^9^), no improvements in RT are observed after rest^4,6,10–13^. Second, there are improvements in RT over some rest intervals but not others^2,3^. For example, a rest period of five minutes, like the one used in the current study, shows an RT improvement. However, if that rest is increased to four hours, there is no improvement^2^. Third, RT improvements after a rest period greater than five minutes only occur in “massed” training conditions when the on-task trial time is 30 s, and not in “spaced” conditions when it is 10 s, despite the total amount of on-task time being equated^4,10^. The consolidation account fails to explain these phenomena.

On the other hand, the accrual and dissipation of RI may be sufficient to explain those phenomena, without the need to infer offline facilitating consolidation. All else held constant, the longer a motor task continues, the greater the RI build-up, resulting in progressively slower RTs. During breaks between trials, RI dissipates. The longer the break, the greater the expected dissipation^4,9,10,12^. Thus, long on-task trial times and short breaks between trials should yield the largest build-up of RI over trials, and consequently, the largest post-rest RT improvement due to RI dissipation. Conversely, short on-task trial time and a long break period will yield the smallest build-up of RI over trials and the smallest post-rest RT improvement.

Those RI effects have not been considered in several recent studies, nor in many past studies in which offline facilitating consolidation has been inferred. This raises the possibility that there is no facilitating consolidation. Rather, it may be that learning occurs concurrently with performance and that dissipation of reactive inhibition creates illusory learning during breaks. The main goal of this work was to test the sufficiency of that alternative account. We investigated whether the amount of post-rest RT improvement that is observed over variations in trial time (10 s vs. 30 s) and break time between trials (10 s vs. 30 s) can be explained by an RI account, without invoking a facilitating consolidation process.

The facilitating consolidation account has two possible interpretations. The first and simpler interpretation is that consolidative processes only occur during the post-training rest period^2^. Thus, as long as the total amount of both training time (the amount of online learning) and the rest periods (amount of consolidation) are equated over groups, then both the amount of consolidation and the associated RT improvement after rest will be the same over groups. The second interpretation has arisen from recent evidence that consolidation may also occur during short breaks between trials and that all learning occurs during those breaks^7,8^. In this interpretation, groups with more frequent and longer breaks will have undergone more consolidation by the end of training. Further, if we assume that there is a finite amount of facilitating consolidation that can occur over the time course of the experiment, consolidation during breaks may reduce the amount of additional consolidation that occurs during the post-training rest period. This assumption has not been made previously in the literature. This version of the consolidation account and the RI account make similar predictions for the amount of post-rest improvements: the smallest post-rest improvement should occur in the 10 s on, 30 s break group (for which there are 1050 s of cumulative break time during practice; 35 breaks at 30 s per break) and largest post-rest improvement in the 30 s on, 10 s break group (for which there are 110 s of cumulative break time; 11 breaks at 10 s per break).

## Results

### Reactive inhibition

To confirm the presence of RI in the 30 s on-task trial groups, we divided each 30 s trial into three consecutive 10 s bins^4,10^. We then compared mean RTs between the first and third bins. A paired-samples *t*-test, averaged over all practice trials, yielded evidence of RI in both the 30 s break group, *t*(36) = −3.42, *p* = 0.0016, *d* = −0.56, and the 10 s break group, *t*(40) = −3.26, *p* = 0.0023, *d* = −0.51. For the 10 s on-task trial groups, we split the 10 s trials into three 3.33 s bins. A paired-samples *t*-test, averaged over all practice trials, yielded evidence of RI in both the 30 s break group, *t*(38) = −8.2, *p* < 0.0001, *d* = −1.32 and the 10 s break group, *t*(41) = −6.3, *p* < 0.0001, *d* = −0.98.

### Post-rest improvement

**Fig. 1:**
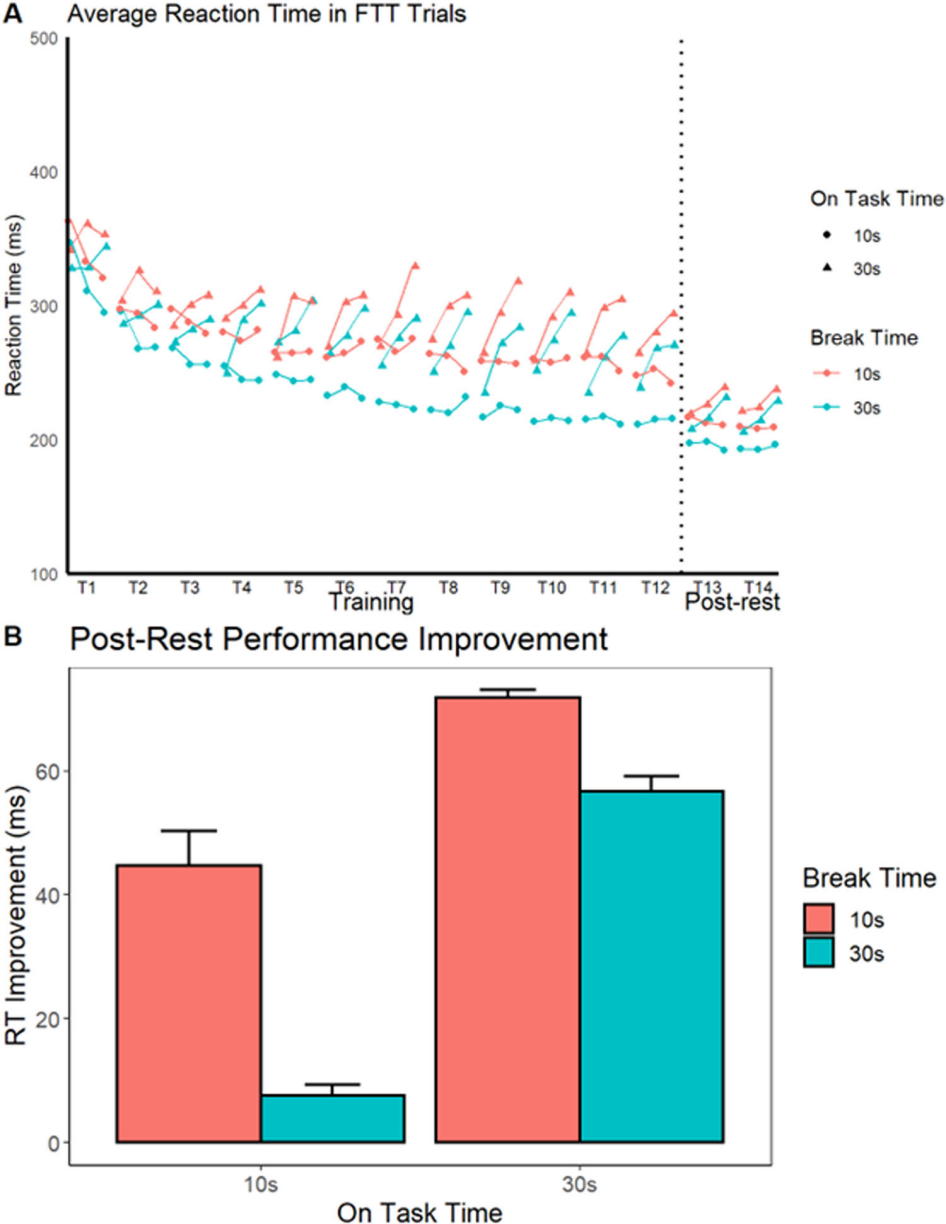
FTT skill learning and post-rest improvement. **A** Each point represents 10 s of on-task trial time. In the 30 s on-task trial time condition, the triangles connected by lines are not separated by breaks. In the 10 s on-task trial time condition, each circle is separated by a break, even if the line connects them. **B** The *y*-axis shows the amount of RT improvement after the rest. The *x*-axis indicates the amount of on-task trial time, whereas the color indicates the amount of break time. Holding the amount of on-task trial time constant (*x*-axis), break time has a strong effect on the amount of RT improvement. When the break time (color) is held constant, on-task trial time also has a strong effect on RT improvement. Error bars in standard error.

With the presence of RI confirmed, we investigated how the amount of on-task time and break time affected the post-rest improvement. As in Brawn et al. (2011), we compared the RT means of the last two training trials (11 and 12) with post-rest trials (13 and 14). A 2 × 2 mixed-factors Analysis of Variance (ANOVA) revealed a significant effect of break time on the post-rest improvement, *F*(1, 155) = 14.49, *p* < 0.001, η^2^ = 0.08 (Fig. 1b), as well as a significant effect of on-task trial time, *F*(1, 155) = 5.79, *p* < 0.01, η^2^ = 0.04 (Fig. 1b). There was no significant interaction between the two factors, *F*(1, 155) = 1.217, *p* = 0.272, η^2^ = 0.007. The same results were found when analyzing the number of correctly completed sequences between the last two training trials and the post-rest trials. The ANOVA revealed a significant effect of break time, *F*(1, 155) = 9.23, *p* = 0.0027, η^2^ = 0.04, as well as a significant effect of on-task trial time, *F*(1, 155) = 85.38, *p* < 0.001, η^2^ = 0.34, with no significant interaction, *F*(1, 155) = 1.722, *p* = 0.19, η^2^ = 0.007.

To investigate if the last two training trials (11 and 12) and post-rest trials (13 and 14) had significantly different RTs within each group, we ran four paired-samples *t*-tests. The 30 s on-task trial, 30 s break group showed a significant decrease in RT, *t*(36) = −3.97, *p* = 0.0003, *d* = −0.65, as did the 30 s on-task trial, 10 s break group, *t*(40) = −9.01, *p* < 0.0001, *d* = −1.41. In contrast, the 10 s on-task trial, 30 s break group showed no evidence of RT decrease, *t*(38) = −0.7, *p*-value = 0.49, *d* = −0.11 whereas the 10 s on-task trial, 10 s break group did, *t*(41) = −7.9, *p* < 0.0001, *d* = −1.22. The null post-rest result for the 10 s on-task trial, 30 s break group suggests that a 30 s break was sufficient to resolve most if not all of the RI build-up that occurred on each trial.

## Discussion

We investigated how the post-rest RT improvement is moderated by on-task trial time and break time. We found that both factors significantly affect the post-rest improvement. The longer the trial and the shorter the breaks, the greater the post-rest improvement, and vice-versa. Those results, along with the clear build-up of RI within trials, are fully consistent with our RI account. This account assumes that learning is concurrent with performance, RI builds during continuous performance, and that it dissipates gradually during breaks.

The simpler interpretation of the consolidation hypothesis predicted that only the amount of total training and the length of the rest interval will affect the post-rest improvement. Because both of those factors were held constant across the four groups, that interpretation predicted null effects, which were not observed. An alternative interpretation of the consolidation hypothesis predicts that consolidation occurs during the much shorter breaks between trials^8^, and that the greater the consolidation during the breaks will result in less consolidation during the rest period (although this latter prediction has not been previously hypothesized in the literature). That prediction is also consistent with the observed post-rest improvement over groups.

Our findings provide the first systematic evidence that an RI-based model assuming online learning and no offline facilitating consolidation can explain motor sequence learning and performance in the context of short breaks and rest periods. Although the revised consolidation account with an additional assumption can also explain those post-rest improvement effects, the RI account has two advantages. First, RI and its dissipation during breaks is clearly a necessary factor in understanding motor performance, whereas facilitating consolidation does not appear to be necessary. Second, our finding that the post-rest RT improvement was negligible and non-significant in the 10 s on-task trial, 30 s break group is not surprising in light of the RI model, given that RI has long been understood as a short-lasting phenomenon^4,9,10,12^. In contrast, there is no precedent in the literature suggesting that facilitating consolidation can be exhausted by a series of 30 s breaks between trials, such that no additional facilitating effect occurred during a subsequent 300 s rest period. Finally, in our 10 s on-task trial and 10 s break group that is analogous to groups used in two recent studies^7,8^, we found evidence of RI, both within trial and across the rest period. This raises the possibility that the offline facilitating consolidation that the authors of those studies inferred in fact reflects solely the dissipation of RI.

This study was not designed to estimate the accrual and dissipation rates of the RI, but we can gain some insight based on the non-significant post-rest RT improvement for the 10 s on-task, 30 s break group, whereas there was a statistically significant post-rest improvement for the other three groups. Within the RI theoretical framework, the null effect in the 10 s on-task trial, 30 s break group indicates that 30 s is sufficient to fully resolve the RI that builds over 10 s trial. Conversely, we know that 10 s of break between 10 s on-task trials is insufficient. Hence, for the case of 10 s on-task trial time at least, RI resolves at a rate that is somewhere between one and three times smaller than the rate at which it accrues. More research is needed to understand what the exact relative rate is and whether it is a constant over different on-task time periods.

In conclusion, the RI account is sufficient to explain the post-rest improvements after a 300 s rest. This finding reinforces the claim that the accrual and dissipation of RI is a critical factor for understanding motor learning and performance over short time scales (for related conclusions in the case of implicit sequence learning, see Török et al., 2017), whereas facilitating consolidation may not be.

## Methods

### Participants

All participants were right-handed. Thirty-seven participants were in the 30 s on, 30 s break group (age = 20.16, F = 67.6%). Thirty-nine participants were in the 10 s on, 30 s break group (age = 20.31, F = 79.5%). Forty-two participants were in the 30 s on, 10 s break group (age = 21.07, F = 79.5%). Forty-four participants were in the 10 s on, 10 s break group (age = 20.54, F = 75%). One participant was removed from the 30 s on, 10 break group and two participants were removed from the 10 s on, and 30 s break group due to corrupted data. Participants provided informed consent via button press. All procedures were approved by the institutional review board of the University of California, San Diego.

### Experimental design and procedure

**Fig. 2:**
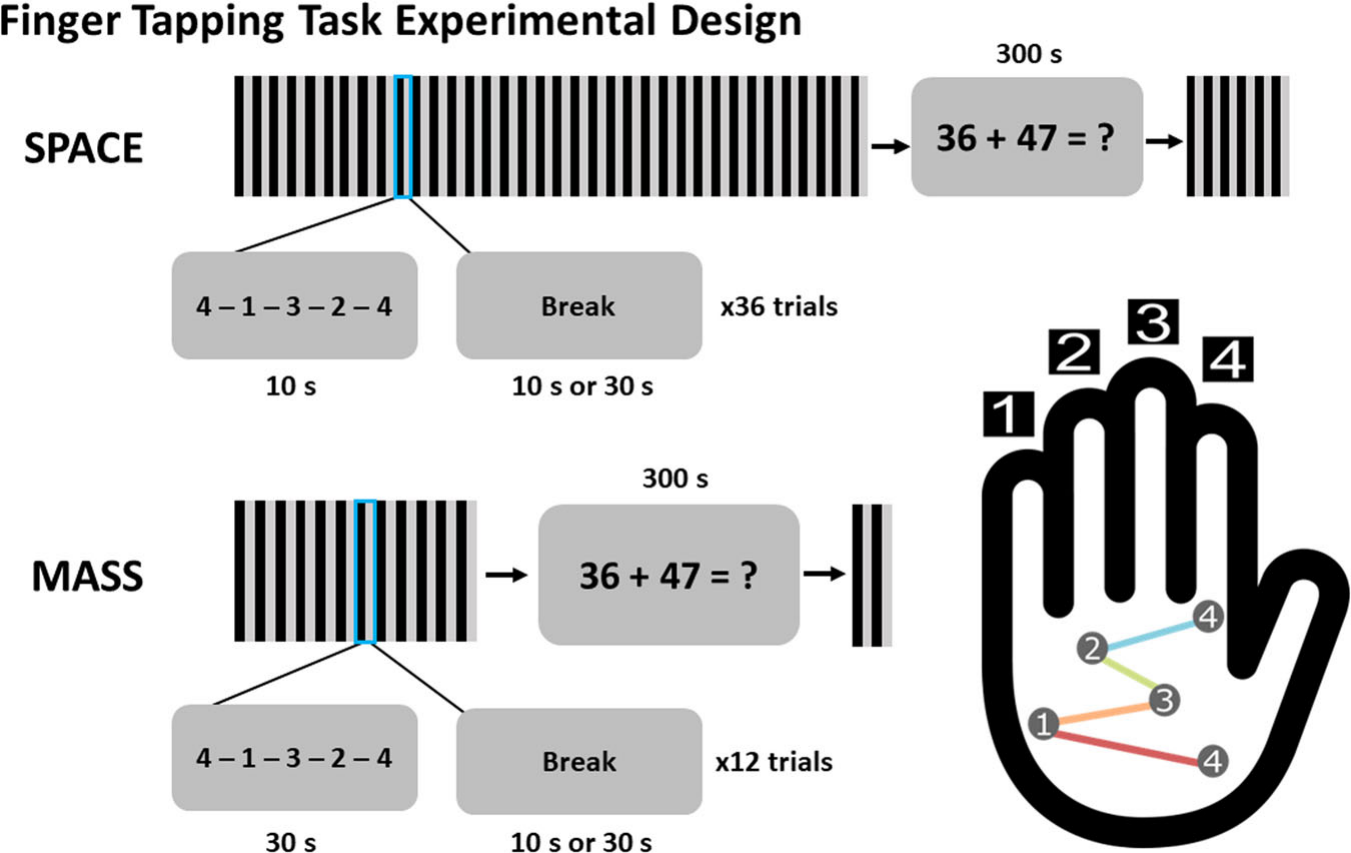
Finger-tapping task. Participants learned a motor sequence over one session. They were instructed to repeatedly type a sequence, 41324, with their non-dominant left hand as fast and as accurately as possible. Keypress 4 was performed with the index finger, keypress 3 with the middle finger, keypress 2 with the ring finger, and keypress 1 with the pinky finger. Participants trained for a total of 360 s in either 10 s or 30 s trials. In between practice trials were either breaks of 10 s or 30 s. After training, participants performed 300 s of double digit addition. They were then tested on the practiced sequence for 60 s with the same trial and break lengths during training.

Participants performed a standard finger-tapping-task where they repeated the sequence, 4-1-3-2-4, as quickly and accurately as possible with their non-dominant left hand^14^. A 2 × 2, between-participant design was used, with factors of Trial Length (10 or 30 s) and Break Period between trials (10 s or 30 s). After the 360 s of on-task training, there was a 300 s rest where participants performed a distraction task of double-digit addition. Afterwards, they performed 60 s of test trials with breaks in between in the same conditions that they trained on (Fig. 2).

### Statistical analysis

The first completed sequence of each trial was considered a warm-up trial and was removed prior to data analysis. RT was defined as the time between temporally adjacent keypresses, where the first keypress RT for a trial was the time since the last keypress of the preceding trial. Keypresses were logged as ‘KEYUP’ events in JavaScript. This event registers the keypress once the key has been released. The post-rest RT improvement was defined as the as the difference between mean RT of the last two training trials (11 and 12) and the mean RT of the post-rest trials (13 and 14).

## Data Availability

All data and code (stimuli and analyses are available online (https://osf.io/khaqv/). Further information and requests for resources should be directed to and will be fulfilled by the corresponding author, TCR (trickard@ucsd.edu).
